# Surveillance of Food- and Smear-Transmitted Pathogens in European Soldiers with Diarrhea on Deployment in the Tropics: Experience from the European Union Training Mission (EUTM) Mali

**DOI:** 10.1155/2015/573904

**Published:** 2015-10-11

**Authors:** Hagen Frickmann, Philipp Warnke, Claudia Frey, Salvatore Schmidt, Christian Janke, Kay Erkens, Ulrich Schotte, Thomas Köller, Winfried Maaßen, Andreas Podbielski, Alfred Binder, Rebecca Hinz, Benjamin Queyriaux, Dorothea Wiemer, Norbert Georg Schwarz, Ralf Matthias Hagen

**Affiliations:** ^1^Department of Tropical Medicine at the Bernhard Nocht Institute, German Armed Forces Hospital of Hamburg, 20359 Hamburg, Germany; ^2^Institute for Medical Microbiology, Virology and Hygiene, University Medicine Rostock, 18057 Rostock, Germany; ^3^Sub-Department VI.2, Department A, Military Medical Command, 56070 Koblenz, Germany; ^4^Department II (Veterinary Medicine), Central Institute of the German Armed Forces Medical Service Kiel-Kronshagen, 24119 Kiel-Kronshagen, Germany; ^5^Deployment Health Surveillance Capability/NATO MilMed COE, 80637 Munich, Germany; ^6^Department of Infectious Disease Epidemiology, Bernhard Nocht Institute for Tropical Medicine Hamburg, 20359 Hamburg, Germany

## Abstract

*Introduction*. Since 2013, European soldiers have been deployed on the European Union Training Mission (EUTM) in Mali. From the beginning, diarrhea has been among the most “urgent” concerns. Diarrhea surveillance based on deployable real-time PCR equipment was conducted between December 2013 and August 2014. *Material and Methods*. In total, 53 stool samples were obtained from 51 soldiers with acute diarrhea. Multiplex PCR panels comprised enteroinvasive bacteria, diarrhea-associated *Escherichia coli* (EPEC, ETEC, EAEC, and EIEC), enteropathogenic viruses, and protozoa. Noroviruses were characterized by sequencing. Cultural screening for Enterobacteriaceae with extended-spectrum beta-lactamases (ESBL) with subsequent repetitive sequence-based PCR (rep-PCR) typing was performed. Clinical information was assessed. *Results*. Positive PCR results for diarrhea-associated pathogens were detected in 43/53 samples, comprising EPEC (*n* = 21), ETEC (*n* = 19), EAEC (*n* = 15), Norovirus (*n* = 10), *Shigella* spp./EIEC (*n* = 6), *Cryptosporidium parvum* (*n* = 3), *Giardia duodenalis* (*n* = 2), *Salmonella* spp. (*n* = 1), Astrovirus (*n* = 1), Rotavirus (*n* = 1), and Sapovirus (*n* = 1). ESBL-positive Enterobacteriaceae were grown from 13 out of 48 samples. Simultaneous infections with several enteropathogenic agents were observed in 23 instances. Symptoms were mild to moderate. There were hints of autochthonous transmission. *Conclusions*. Multiplex real-time PCR proved to be suitable for diarrhea surveillance on deployment. Etiological attribution is challenging in cases of detection of multiple pathogens.

## 1. Introduction

Diarrhea due to food- and smear-transmitted pathogens remains a scourge of military deployments in tropical settings. Potential deleterious consequences of this problem were first described more than 3,000 years ago in the Old Testament (Deuteronomy 23: 9–14), detailing basic hygiene procedures such as isolation of infected soldiers and eradication of infectious material in times when there was no concept of microbial pathogens. Recently described deployment-associated outbreaks of gastrointestinal infections confirm the relevance of the issue [1, 2].

As previously described [3], infections with food- or waterborne enteric pathogens can be efficiently prevented by elaborate hygiene precautions in military field camps on deployment. In German field camps, these precautions comprise compliance with European general principles and requirements of food law, European procedures regarding food safety (Regulation EC number 178/2002), and the German Food and Feed Law (“Lebensmittel- und Futtermittelgesetzbuch,” LFBG); production and delivery of food and drinking water by German soldiers or under their direct supervision whenever possible; implementation of HACCP (hazard analysis and critical control points) systems from delivery to disposal, including cleaning and disinfection measures in conjunction with food production as well as handling procedures by operators of dining and water treatment facilities; and instructions in accordance with the German infection prevention law (“Infektionsschutzgesetz,” IfSG) for military and civilian staffs of facilities supplying food and water. Military public health officials such as veterinarians or hygiene officers are in charge of all food and drinking water control procedures. Laboratory surveillance, generally focused on infectious and noninfectious threats that might endanger the mission, is carried out with samples of delivered and prepared food and treated water prior to release and also includes the screening of local staffs for pathogens according to relevant directives from the hygiene department. Generally, all deployed soldiers are by order forbidden to consume other than safety-approved food and drinking water from the country of deployment. Such safety approval requires that local producers are audited and controlled by military food specialists.

If such high hygiene standards can be maintained, infection rates with enteric pathogens are comparably low to those in Germany as shown for chronic infestations with enteropathogenic protozoa in German soldiers returning from German military field camps in various subtropical and tropical deployment sites [3]. If these standards cannot be maintained for logistic reasons, for example, during small or multinational deployments, infection rates increase [3]. Further, in spite of training and education in basic hygienic measures that are implemented before deployment, soldiers may tend to noncompliance if the temptation of appealing local foods coincides with monotonous food in the field canteen or perhaps delivery problems during the first phases of deployment. A previous study described noncompliance problems with antimalarial chemoprophylaxis on deployment [4].

German hygiene standards for military field camps cannot always be maintained during multinational military operations. German soldiers have, for example, participated in the European Union Training Mission (EUTM) in Mali since March 2013, with the field camp in Koulikoro near the capital Bamako as the major site of deployment. Diarrhea has been among the most “urgent” infectious concerns from the beginning. Consequently, deployable real-time PCR equipment was transferred to Koulikoro to study the molecular epidemiology of diarrhea in the camp from December 2013 until March 2014. Subsequently, the surveillance of food- and smear-transmitted pathogens was continued until August 2014 by transferring sample material to Germany. Here we describe results that were obtained in a 9-month observation period.

## 2. Materials and Methods

### 2.1. Study Population

Between the 49th calendar week in December 2013 and the 34th calendar week in August 2014, microbiological surveillance was carried out on European soldiers with acute diarrhea deployed in Mali in the course of the European Union Training Mission (EUTM). Diarrhea was defined as the deposition by the subject of amorphous stools and distinguished according to frequency as <3 or ≥3 stools per day. Accordingly, low-frequency diarrhea was considered as of potential infectiological relevance as well. The field doctor in charge subjectively decided whether or not he or she considered the symptoms of the patients to be relevant and incapacitating enough for an inclusion into the surveillance. All diarrhea patients were asked by the field doctor in camp Koulikoro to provide a sample of native stool for molecular and cultural diagnostic analysis. Investigations of diseased local military or civilian personnel were not part of the surveillance.

In addition to providing the samples, the field doctor collected data on diarrhea patients in a standardized way on the delivery note that was sent together with the sample. The items on these notes were as follows: age, gender, nationality, mode of food intake, site of deployment, stool frequency, stool consistency as described by the patient, accompanying symptoms, presence of fever, type of antimalarial prophylaxis, vaccination status against cholera and typhoid fever, medication with antibiotic drugs, any previous stays in high-endemicity settings regarding diarrhea (including Afghanistan, Bahrain, Bolivia, Colombia, Kosovo, Lebanon, and Turkey [3]), and participation in counseling regarding hygiene on tropical deployments prior to the onset of diarrhea. In addition, stool consistency of the samples provided was assessed by the analyzing laboratory technician.

While providing stool samples and thus participating in the surveillance were voluntary, all cases of diarrhea among EUTM personnel that were registered by the field doctor were further routinely reported to the Deployment Health Surveillance Capability (DHSC) of the North Atlantic Treaty Organization (NATO) Military Medicine Center of Excellence. Again, the diagnosis diarrhea was based on the field doctor's subjective decision whether or not he or she considered the symptoms of the patients to be relevant and incapacitating. These data and the number of deployed EUTM forces were used to calculate a weekly incidence rate as a passive surveillance procedure. DHSC reports were compared with the number of patients that were included in the surveillance to estimate the achieved coverage.

### 2.2. Laboratory Testing

From December 2013 to March 2014, collected stool samples were analyzed by PCR directly in the laboratory of the field hospital in Camp Koulikoro in Mali. From April until August 2014, the samples were frozen at −20°C and shipped to Germany for further investigation. All culture-based diagnostic approaches were performed in Germany.

#### 2.2.1. Sample Preparation

Prior to PCR analysis, nucleic acids were extracted from all stool samples using the Qiamp stool kit (Qiagen, Hamburg, Germany). The nucleic acid extraction was performed as described by the manufacturer.

#### 2.2.2. PCR Testing

All samples were analyzed with a panel of real-time multiplex PCRs comprising two in-house protocols. One of those in-house PCR tests targets the invasive enteropathogenic bacteria* Salmonella* spp.,* Shigella* spp./enteroinvasive* Escherichia coli* (EIEC),* Campylobacter jejuni*, and* Yersinia* spp. [5, 6]. The diagnostic reliability of this procedure has been shown previously in comparison with cultural approaches [5]. The other in-house PCR, which is used for routine diagnostic analyses by the German National Reference Centre for Tropical Diseases Bernhard Nocht Institute Hamburg, amplifies DNA of the enteropathogenic protozoa* Entamoeba histolytica*,* Giardia duodenalis*,* Cyclospora cayetanensis*, and* Cryptosporidium parvum* [3, 6, 7]. The previously described primer-probe-sets for* Entamoeba histolytica*,* Giardia duodenalis*, and* Cryptosporidium parvum* were complemented by a primer-probe-set for* Cyclospora cayetanensis* that was adapted from a previous publication [7]. No further changes to the described protocols [3, 5, 6, 8] were applied. The primer-probe-sets used are detailed in Table 1.

In addition to the in-house multiplex real-time PCR assays described above, three commercial RidaGene (R-Biopharm, Darmstadt, Germany) PCR kits: “EAEC,” “EHEC-EPEC,” and “ETEC-EIEC,” were applied targeting enteroaggregative* E. coli* (EAEC), enterohemorrhagic* E. coli* (EHEC), enteropathogenic* E. coli* (EPEC), enterotoxic* E. coli* (ETEC), and* Shigella* spp./EIEC. Finally, analyses using the commercial Fast-track Diagnostics (Sliema, Malta) PCR kit “viral gastroenteritis” targeting Norovirus genogroups I (G1) and II (G2), Astrovirus, Rotavirus, Adenovirus, and Sapovirus were added.

Phocid herpesvirus DNA was chosen as the target for inhibition control PCR. The procedure was performed as described previously [8–10]. The primer-probe-set is presented in Table 1.

#### 2.2.3. Norovirus Sequencing

Samples testing positive by Norovirus genogroup I (GI) or II (GII) PCR were subjected to further sequence analysis. In detail, a 213-base-pair (bp) part of the open reading frame 1 (ORF1) was amplified using degenerate multiplex primers MON432/MON434 (GI) and MON431/MON433 (GII) as described [11]. Amplified DNA was sent for sequencing (Seqlab, Göttingen, Germany) after nucleic acid gel extraction and cleanup with a QIAquick Gel Extraction Kit (QIAGEN, Hilden, Germany). Sequence assembly and analysis were performed with the software DNAStar Lasergene 12.1 genomics suite (DNASTAR Inc., Madison, WI, USA). Subsequent genotyping was based upon the Norovirus genotyping tool as described [12].

#### 2.2.4. Cultural Growth, Identification, and Resistance Testing

If sufficient stool material was available, cherry-pit-sized volumes were used for broth enrichment in thioglycolate broth (Heipha, Eppelheim, Germany). This was the case for a total of 48 out of 53 samples. Incubation was performed for 16–24 hours at 37°C. Broth enrichment increases the yield of ESBL-expressing bacteria after swabbing, for example, by a factor of 2 in upper respiratory tract samples [13]. Subsequently, 10 *μ*L preincubated broth was cultured on Brilliance ESBL selective agar (Oxoid, Basingstoke, UK). This agar is made for selective growth of ESBL-positive Enterobacteriaceae. Sensitivity of 94.9–97.9% and specificity of 95.7–100% have been described for Brilliance ESBL selective agar [14, 15]. Agar plates were incubated at 37°C for 40–48 hours. All colonies that looked suspicious for Enterobacteriaceae (blue, green, and brown colonies) were isolated, while suspected Gram-negative nonfermentative rod-shaped bacteria (i.e., yellow or yellowish-brown or greenish-brown colonies) were discarded. All isolates were frozen at −80°C in Microbank tubes (Pro-Lab Diagnostics, Bromborough, UK) until further assessment.

Identification was performed by VITEK-II GN-cards (bioMérieux, Marcy-l'Étoile, France) and matrix-assisted laser-desorption-ionization time-of-flight mass spectrometry (MALDI-TOF-MS) using a Shimadzu/Kratos “AXIMA Assurance” MALDI-TOF mass spectrometer (Shimadzu Germany Ltd., Duisburg, Germany) [16]. For MALDI-TOF analyses, isolates were prepared using alpha-cyano-4-hydroxycinnamic acid (bioMérieux) as matrix. Spectral fingerprints were analyzed using Vitek MS IVD V2, database MS-CE version CLI 2.0.0 (bioMérieux). Automated antibiotic susceptibility testing was performed with VITEK-II AST-N263-cards (bioMérieux). In case of uncertain results, E-testing (bioMérieux) was added. Interpretation of resistance testing results was based on the EUCAST guideline (version 4.0, 2014, http://www.eucast.org/fileadmin/src/media/PDFs/EUCAST_files/Breakpoint_tables/Breakpoint_table_v_4.0.pdf).

#### 2.2.5. Rep-PCR Typing of Extended-Spectrum Beta-Lactamase Positive* E. coli*

All confirmed ESBL-positive* E. coli* strains from ESBL selective agar were grown overnight in brain heart infusion broth. DNA was extracted using the MoBio UltraClean Microbial DNA Isolation Kit (Mo Bio Laboratories Inc., Carlsbad, CA, USA). Purified DNA samples were amplified using the DiversiLab* Escherichia* DNA fingerprinting kit (reference number: 410 980, bioMérieux) on a T-personal thermal cycler (Biometra, Göttingen, Germany). Rep-PCR products were detected by chip-based DNA separation on an Agilent 2100 Bioanalyzer (Agilent Technologies Inc., Santa Clara, CA, USA). All techniques were executed according to the manufacturers' instructions.

Documentation and band-pattern analysis were performed using the DiversiLab software version 3.3 (bioMérieux). A correlation cutoff of 95% for confirmation or exclusion of clonal identity of analyzed strains was applied as recommended by the manufacturer. All library entries were analyzed in duplicate.

### 2.3. Ethics

The surveillance described here was ordered as a preventive medical procedure by the German commanding hygiene officer of the EUTM Mali mission. Respective orders are legally covered by the self-administrative rights “Eigenvollzugskompetenz” of the German Armed Forces Medical Service regarding infectious disease prevention and control “Infektionsschutz”. Collected data were assessed anonymously, thus avoiding any violation of §25 of the Declaration of Helsinki (DoH/Oct 2008) or national data protection laws “Bundesdatenschutzgesetz”.

## 3. Results

### 3.1. Frequency of Diarrhea and Coverage

Based on the reports of the field doctors to the NATO Deployment Health Surveillance Capability (DHSC), an average weekly incidence of diarrhea among EUTM soldiers of 5.8 patients per week was calculated. The average number of deployed soldiers at risk was 480; the resulting average weekly incidence rate per 1,000 soldiers was 12.1. During the surveillance period of 37 weeks, a total of 53 stool samples from 51 EUTM soldiers with diarrhea were collected, resulting in an average of 1.4 cases per week. Accordingly, the coverage of the surveillance was about 24.1% of the registered diarrhea cases.

### 3.2. Diagnostic Results

Positive PCR results for diarrhea-associated pathogens could be detected in 43/53 patient samples. The five quantitatively dominating pathogens were EPEC (*n* = 21), ETEC (*n* = 19), EAEC (*n* = 15), Norovirus (*n* = 10), and* Shigella*/EIEC (*n* = 6), followed by* Cryptosporidium parvum* (*n* = 3),* Giardia duodenalis* (*n* = 2),* Salmonella* spp. (*n* = 1), Astrovirus (*n* = 1), Rotavirus (*n* = 1), and Sapovirus (*n* = 1). Of note, both detections of* Giardia duodenalis* were in the same patient, so copy-strain assessment occurred here. Median and mean cycle threshold (Ct) values as well as calculated standard deviations (SD) are given in Table 2. Of note, the lowest Ct values were detected for bacteria. Ct-values for* Shigella* spp./EIEC varied considerably depending on the primer-probe composition used, with lowest Ct-values in the RidaGene ETEC/EIEC kit and highest in the in-house approach.

DNA of two and more pathogens was detected in 23 of the samples, of three and more pathogens in 11 samples, of four and more pathogens in 2 samples, and of as many as five pathogens in 1 sample. Measured Ct values for the respective cases are given in Table 3. In several cases, low Ct-values are measured for more than one pathogen.

### 3.3. Norovirus Genotyping

Sequence analysis and genotyping verified the detection of Norovirus GII in six out of seven initially PCR-positive cases. The procedure failed for the seventh Norovirus GII detection and for all three cases positive for Norovirus GI. At least two out of three Norovirus GI-positive cases were confirmed by a separate real-time RT-PCR [17, 18] (data not shown).

Genotyping of the six sequenced Norovirus G2 strains revealed GII.P7 in four instances. Three out of those four strains showed identical sequences, suggesting either nosocomial transmission or a common source of infection. Epidemiological assessment showed that the respective samples were collected from three patients within a single week, making a mini-outbreak highly likely. In two out of six instances, Norovirus GII.P16 and GII.P4 var New Orleans were identified, respectively.

The underlying sequence information has been deposited and is freely accessible via http://www.rivm.nl/mpf/norovirus/typingtool/job/1197792283/.

### 3.4. Cultural Approach

From 13 out of the 48 analyzed stool samples, ESBL-positive Enterobacteriaceae were isolated by thioglycolate broth enrichment with subsequent growth on ESBL selective agar. From 12 samples, ESBL-positive* Escherichia coli* were isolated with proof of more than one strain in two instances. The total number of ESBL-positive* E. coli* strains was 15. One of those strains was identified as EAEC by RidaGene PCR. An ESBL-positive* Klebsiella pneumoniae* strain was isolated from another sample.

For the 13 samples containing ESBL-positive Enterobacteriaceae, sensitivity against nonpenicillin, noncephalosporin antibiotics was determined by VITEK-II- and E-test-based resistance testing. If several ESBL-positive* E. coli* were simultaneously isolated, the most resistant strain with the resulting highest risk of selection under antibiotic pressure was chosen. Sensitivity for carbapenems, tigecycline, and fosfomycin was shown in all 13 cases; in 12 for nitrofurantoin, which is only suitable for urinary tract infections; in 11 for fluoroquinolones; and in 10 for gentamicin. Resistance against trimethoprim/sulfamethoxazole, which is frequently encountered in tropical settings, was demonstrated in all 13 cases.

### 3.5. Rep-PCR of ESBL-Positive Enterobacteriaceae

**Figure 1:**
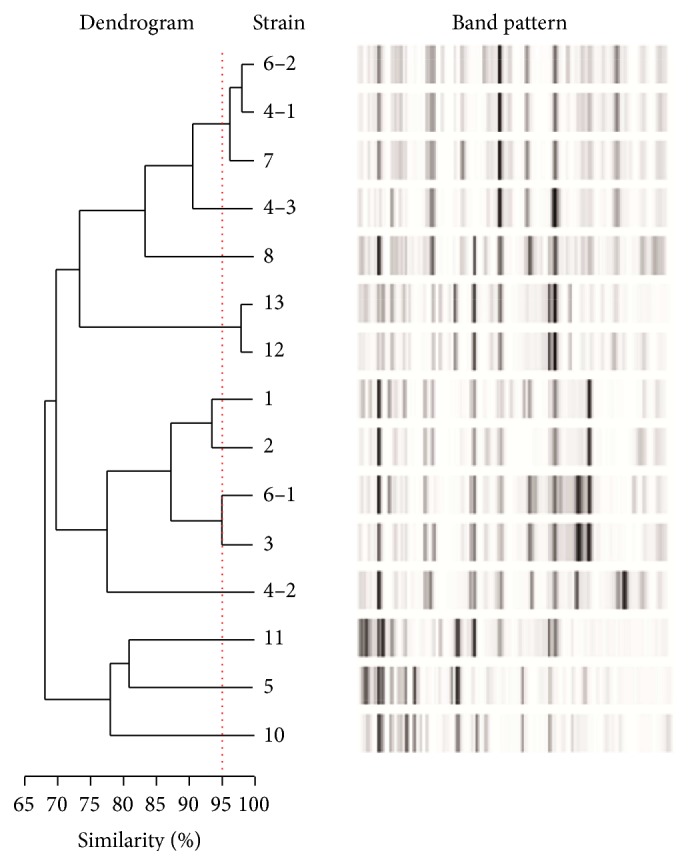
Dendrogram of the DiversiLab typing results of 15 ESBL-positive* E. coli* strains from 12 patients. The strains' labeling consists of the patient number (number before the “–”) and the strain number in case of more than one isolated ESBL-positive* E. coli* strain per patient (number following the “–”). The strain of patient 9 is missing, as the respective ESBL-positive isolate was not* E. coli* but* K. pneumoniae*. Three clusters of clonal identity beyond the 95%-similarity cutoff (dotted red line) are detectable.

In total, 15* E. coli* strains from 12 patients were subjected to rep-PCR-based DiversiLab typing. Within the 95% cutoff range for clonal identity, three clonal clusters comprising 7 strains from 6 patients were observed (Figure 1). This suggests the presence of common sources of infection or nosocomial transmission within the camp. One patient was even colonized by* E. coli* strains from two different clusters (Figure 1). For the remaining 8 strains from 6 patients, clonal identity was excluded.

### 3.6. Clinical and Epidemiological Assessment

Clinical data were provided for 49 patients. The completeness of clinical data varied: missing data are characterized as “no data” in the tables in the following. In those 49 diarrhea patients, bacterial pathogens were detected in 34 instances, viral pathogens in 12 instances, and parasitic pathogens in 3 instances. No enteric pathogen was detectable in 9 out of these 49 patients with the applied procedures. DNA of more than one enteric pathogen was detectable in 23 of the soldiers with acute diarrhea.

#### 3.6.1. Age, Gender, Nationality, Sites of Deployment, and Previous Stays in High-Endemicity Regions for Diarrhea-Associated Pathogens

No particular distribution pattern of bacterial, viral, and parasitic enteric pathogens was observed with respect to gender and nationality of the soldiers with diarrhea. Soldiers who were younger than 30 years of age showed coinfections with multiple enteric pathogens less frequently than older soldiers. Of note, all assessed diarrhea patients who were deployed to Bapho were infected by viral pathogens. Previous stays in high-endemicity settings did not have any notable effects on the acquisition of bacterial, viral, and parasitic pathogens (Table 4).

#### 3.6.2. Mode of Food Intake

As few as 2 out of 15 diarrhea patients who claimed to have eaten exclusively in the field kitchen were free of enteric pathogens in the PCR analyses. Bacterial pathogens dominated in this group. Soldiers who restricted their diet to hotel food were prone to both bacterial and viral enteric infections. There was not a single diarrhea patient in this assessment who had eaten field rations alone prior to the onset of diarrhea (Table 5).

#### 3.6.3. Stool Frequency and Consistency

A small proportion of 11 patients showed less severe symptoms with fewer than three unformed stools per day. Of note, no enteric pathogens were detected in only one of these patients. There was no obvious distribution pattern of bacterial, viral, or parasitic pathogens in these less severe diarrhea cases.

The observed stool consistency of collected samples, as reported by the laboratory technician, was considerably less unformed than the reported stool consistency. A total of 13 out of 49 stool samples were already hard at the time of sample collection, suggesting that the symptoms had already improved. In contrast, no patient reported formed stools to the field doctor. Only 3 out of these 13 formed stools were without detectable pathogen DNA at the time of assessment (Table 6).

#### 3.6.4. Accompanying Symptoms including Fever

Light to moderate symptoms including nausea and vomiting, cramps, abdominal pain, and flatulence were frequent in the assessed diarrhea patients. Cramps, abdominal pain, and flatulence were particularly often detectable in diarrhea patients with bacterial infections, while nausea and vomiting were equally likely for both bacterial and viral infections. Only one instance of bloody diarrhea was observed in a patient with* Shigella* spp./EIEC as the only detectable pathogen in stool. Fever was confirmed in only three instances without any detectable association with a particular pathogen group (Table 7).

Of note, the distribution of accompanying symptoms did not change considerably if only the 34 patients who reported ≥3 stools per day were included into the assessment (Table 7).

Only one enteric pathogen per patient was detected in 16 patients who reported accompanying symptoms, comprising 4 cases with STEC, 2 cases with EAEC, 2 cases with EPEC, 2 cases with Norovirus G1, 2 cases with Norovirus G2, 2 cases with* Shigella* spp./EIEC, 1 case with* Cryptosporidium parvum*, and 1 case with Rotavirus, respectively. Bacterial infections were associated with a broad distribution of symptoms (Table 7). As expected, patients infected with enteroinvasive* Shigella* spp./EIEC showed a particularly wide spectrum of symptoms. Norovirus infections and* Cryptosporidium parvum* infections were associated with nausea, vomiting, and abdominal pain, and Norovirus G1 infections also were associated with cramps and flatulence (Table 7).

#### 3.6.5. Antimalarial Prophylaxis, Hygiene Counseling, Vaccination against Cholera and Typhoid Fever, and Medication

Low-dose doxycycline-monohydrate antimalarial prophylaxis at 100 mg/day did not provide any protection against bacterial enteric infections. Among the diarrhea patients under doxycycline prophylaxis, 7 out of 10 were positive for DNA of bacterial enteric pathogens.

The vast majority of diarrhea patients were properly counseled regarding hygiene on tropical deployments and were vaccinated against typhoid fever. Infections with bacterial enteric pathogens were particularly frequent in patients who were vaccinated against cholera and typhoid fever. Use of anti-infective drugs was documented for three patients; all three took rifaximin and one in addition metronidazole. DNA of bacterial enteric pathogens was detectable in only one of these patients; no pathogen DNA was observed in two of them (Table 8).

## 4. Discussion

Risk assessment by standardized monitoring and surveillance of deployed soldiers in subtropical or tropical countries contributes to evaluation of both individual risk and preventive measures. As previously shown, infection risks with enteric pathogens increase if sophisticated hygiene precautions regarding food and drinking water cannot be maintained on military deployments, for example, in the case of small missions [3]. Considering an average clinical incidence of diarrhea between 5% and 7% per 100 per month on military deployments [19], the estimated average weekly incidence rate of 12.1 per 1,000 soldiers for the EUTM forces is not surprising. The slightly lower incidence might be attributable to the comparably good hygiene standards in Camp Koulikoro.

In the surveillance of deployed European soldiers with diarrhea in tropical Mali described here, noninvasive EPEC, ETEC, and EAEC clearly predominated, followed by Norovirus and* Shigella* spp./EIEC, while other invasive bacteria and protozoa were less frequent. The surveillance interval included periods of both dry season and rainy season, when diarrhea is usually more frequent. Multiplex real-time PCR proved to be a suitable platform for the identification of multiple pathogens in parallel assays, thus allowing for a rapid diagnosis with subsequent enforcement of adequate hygiene precautions. Of note, demonstration of pathogen DNA was still possible in the subacute state when the stool consistency had already changed from fluid or mushy to hard.

Diarrhea-associated* E. coli* strains are frequent in tropical settings as previously shown [20–23], so the dominance of EPEC, ETEC, and EAEC is not surprising. Recent studies further stress the importance of enteropathogenic viruses, in particular Norovirus, in tropical settings [21, 24]. Due to its high contagiousness and tenacity [25, 26], Norovirus is particularly prone to causing local outbreaks [27]. Accordingly, its rapid and reliable identification is of use in military deployments to allow for a rapid enforcement of appropriate hygiene precautions.

Norovirus genotyping confirmed the worldwide occurrence of different genotypes that lead to outbreaks under conditions of restricted hygiene. As shown for three patients with identical Norovirus sequences who became symptomatic within a single week, a single source of infection or person-to-person transmission due to low hygienic compliance may easily affect several soldiers on deployment. Moreover, genotype II.4 is a pandemic strain, which has a high potential to cause nosocomial outbreaks [28]. Thus the sequencing results obtained, confirming at least one small outbreak event, stress the importance of rapid Norovirus diagnostics on deployment.

The inability of genotyping in initially positive tested samples is a consequence of the high mutation rate in Norovirus. In contrast, depending on the targeted sequence, false positive results of Norovirus PCR tests may occur [29]. However, this was not confirmed in this study.

The relative lack of enteroinvasive bacteria and enteropathogenic protozoan parasites was consequently associated with predominantly mild to moderate symptoms in diseased soldiers. Only one case of bloody diarrhea in a patient with* Shigella* spp./EIEC as the only detectable pathogen in the stool sample was observed.* Salmonella* spp. was detected in one instance only;* Campylobacter jejuni* was not observed at all. This speaks in favor of the locally practiced food and drinking water hygiene, as enteroinvasive bacteria [30, 31] and enteropathogenic protozoa [32–35] are frequent causes of severe gastrointestinal infections in sub-Saharan Africa.

The frequent occurrence of bacterial diarrhea in soldiers who ate exclusively at the field kitchen suggests autochthonous spread of pathogens [36, 37] within the field camp, for example, by smear infection. A possible reason could be inadequate toilet hygiene. Also, occasional hygiene problems in the field kitchen could not be excluded, because no soldier with diarrhea reported exclusive consumption of standardized field rations, for which the risk of acquiring gastrointestinal infections is virtually zero.

The data regarding the food sources of the infected soldiers should be interpreted with care. As uncontrolled food consumption outside military infrastructure might pose a disciplinary offense, interpretability of respective information on the questionnaire is limited by a reporting bias. Accordingly, it cannot be excluded that a considerable proportion of diarrhea patients who claimed to have exclusively eaten at the field kitchen indeed consumed food from outside the camp as well.

In spite of a reported partial protective effect of cholera vaccination against travelers' diarrhea [38], detection of DNA of enteropathogenic bacteria was particularly frequent in the cholera-vaccinated soldiers.

As a further result, the surveillance impressively demonstrates the potential multicausal etiology of acute diarrhea on tropical deployments, which has to be considered if targeted therapy of a specific identified pathogen fails. Asymptomatic pathogen carriage was not excluded but is unlikely because the deployed soldiers analyzed did not arrive from high-endemicity settings. As is typical for surveillance analyses, no stool samples were collected prior to deployment, which would have allowed for comparison testing; this is an undeniable limitation of the data presented.

The inclusion of patients into the surveillance merely based on the subjective assessment of the local field doctor is a major limitation of the study. This limitation does not allow direct comparisons with studies using standardized definitions of travelers' diarrhea, for example, including stool quality assessments like the Bristol stool scale [39–42]. Of note, the assessment of symptoms of patients with ≥3 stools per day led to similar results as observed for the whole study population. The focus of the surveillance was on patients with gastrointestinal symptoms leading to incapacitation from military duty, not on patients meeting a standard definition of travelers' diarrhea. Therefore, such a nonconventional inclusion strategy was chosen.

PCR is a highly sensitive method for the detection of enteric pathogens in stool, outperforming alternative approaches such as microscopy regarding the detection limit [5, 43]. However, the problem of simultaneously detecting several enteric pathogens by PCR in stool samples in high-endemicity settings for diarrheal disease is a constant stumbling block, because it hinders etiological attribution and subsequent targeted antimicrobial therapy in case of severe disease. Here we could demonstrate that this problem also applies to European soldiers deployed in the tropics.

Quantitative PCR tests have been suggested as useful tools for a more reliable attribution of etiological significance to detected enteric pathogens [44, 45], discriminating active infection from asymptomatic carrier status or shedding of residual pathogen DNA after previous, already cleared infections. However, no generally accepted standards for such quantitative approaches have been established so far. DNA quantification in complex materials like stool samples is further limited by various degrees of PCR inhibition [46]. In this surveillance, low Ct-values, potentially suggesting etiological relevance, were observed for more than one pathogen in several instances. Sufficiently powered future studies will be necessary to evaluate the usefulness of quantitative stool PCR and the definition of reliable cut-off values for the diagnostic routine.

However, etiological attribution is not the only aspect that makes calculated antimicrobial therapy challenging in case of severe diarrhea in soldiers on deployment. Knowledge about the local antimicrobial-resistance situation in diarrhea-associated bacteria is crucial to allow for a tailored antimicrobial therapy. Next to standard recommendations regarding the therapy of acute gastroenteritis [47] and traveler's diarrhea [48–52], the British and U.S. military medical services also intend studies on the optimization of single-dose antibiotic treatment regimens [19].

High rates of colonization with atypically resistant or even multidrug-resistant bacteria in returnees from tropical settings have recently been described [53–55]. Increased colonization with multidrug-resistant bacteria in the tropics can be triggered by prescribing antibiotics for travelers' diarrhea [56, 57]. However, during the International Security Assistance Force (ISAF) mission in Afghanistan, colonization of German soldiers with ESBL-positive Enterobacteriaceae was as low as 5% [58], despite considerably higher colonization rates in Afghan patients. In contrast, nearly every fourth stool sample of European soldiers demonstrated ESBL colonization in our present surveillance during the EUTM Mali deployment. Although rep-PCR suggests a moderate degree of clonal diversity of ESBL-positive strains from Mali, several clonal mini-clusters suggest either fecal contamination of common sources of infection or nosocomial spreading within the field camp.

In persons of weakened immunological state (e.g., after polytrauma on deployment), transition of enteric bacteria through the gut tissue with resulting sepsis may occur. If resistant bacteria enter blood circulation in this way [59–61], antibiotic therapy becomes challenging. The probability of such events rises in case of high colonization rates with resistant bacteria and selective pressure due to antibiotic therapy or prophylaxis. It is a well-documented phenomenon that colonizing resistant bacteria can cause blood stream infections under the selective pressure of antibiotics [62–66].

The high incidence of ESBL-positive Enterobacteriaceae in deployed soldiers in Mali suggests the use of alternative antibiotic drugs in case of systemic infections. According to German recommendations, oxyimino-cephalosporins (e.g., ceftazidime) or aminoacyl penicillin-beta-lactamase combinations (e.g., piperacillin/tazobactam) are appropriate substances for calculated initial therapy of sepsis [67]. However, these substances will fail in case of sepsis due to ESBL-positive Enterobacteriaceae. Furthermore, resistance against the orally administrable fluoroquinolones was observed in several instances, making the use of intravenous reserve substances such as carbapenems unavoidable if severe systemic bacterial infections occur.

Of note, increasing antibiotic resistance has recently been described for diarrhea-associated* E. coli* and* Shigella* spp. as well [68]. In this study, only one ESBL-positive EAEC was isolated.

No efficient procedures for reliable eradication of enteric colonization with ESBL-positive Enterobacteriaceae have been described so far. Accordingly, a high probability of such colonization in returnees from Mali has to be considered both for hygienic reasons and for the choice of antibiotic drugs in case of future systemic infections.

## 5. Conclusions

Real-time multiplex-PCR systems proved to be useful for diarrhea surveillance in the tropical deployment setting, allowing the detection of enteric pathogens in more than 80% of the analyzed stool samples of European soldiers in Mali. However, the frequent detection of DNA of several pathogens in high-endemicity settings impedes the etiological attribution. Noninvasive enteropathogenic bacteria and Norovirus dominated quantitatively and were associated with mild to moderate symptoms. The reported mode of food intake suggests the presence of transmission routes in the field camp.

## Figures and Tables

**Table 1:** Sequences of the primer-probe-sets used in the applied real-time PCR assays [5–9].

Target organism	Forward primer	Reverse primer	TaqMan probe
Multiplex-PCR targeting invasive enteric bacteria			

*Salmonella* spp.	5′-ATT-GTT-GAT-TCA-GGT-ACA-AAC-3′	5′-AAT-TAG-CCA-TGT-TGT-AAT-CTC-3′	5′-CAA-GTT-CAA-CGC-GCA-ATT-TA-3′
*Shigella* spp./EIEC	5′-CAG-AAG-AGC-AGA-AGT-ATG-AG-3′	5′-CAG-TAC-CTC-GTC-AGT-CAG-3′	5′-ACA-GGT-GAT-GCG-TGA-GAC-TG-3′
*Campylobacter jejuni *	5′-CTA-TAA-CAA-CTG-CAC-CTA-CTA-AT-3′	5′-AAG-TGT-AAG-CAC-ACA-AGG-TA-3′	5′-CTT-AAT-AGC-CGT-CAC-CCC-AC-3′
*Yersinia* spp.	5′-GCA-TTA-ACG-AAT-ATG-TTA-GC-3′	5′-ATC-GAG-TTT-GGA-GTA-TTC-AT-3′	5′-CCG-CTT-CCA-AAT-TTT-GTC-AT-3′

Multiplex-PCR targeting enteric protozoa			

*Entamoeba histolytica *	5′-ATT-GTC-GTG-GCA-TCC-TAA-CTC-A-3′	5′-GCG-GAC-GGC-TCA-TTA-TAA-CA-3′	5′-TCA-TTG-AAT-GAA-TTG-GCC-ATT-T-3′
*Giardia duodenalis *	5′-GAC-GGC-TCA-GGA-CAA-CGG-TT-3′	5′-TTG-CCA-GCG-GTG-TCC-G-3′	5′-CCC-GCG-GCG-GTC-CCT-GCT-AG-3′
*Cyclospora cayetanensis *	5′-TAG-TAA-CCG-AAC-GGA-TCG-CAT-T-3′	5′-AAT-GCC-ACG-GTA-GGC-CAA-TA-3′	5′-CCG-GCG-ATA-GAT-CAT-TCA-AGT-TTC-TGA-CC-3′
*Cryptosporidium parvum *	5′-CGC-TTC-TCT-AGC-CTT-TCA-TGA-3′	5′-CTT-CAC-GTG-TGT-TTG-CCA-AT-3′	5′-CCA-ATC-ACA-GAA-TCA-TCA-GAA-TCG-ACT-GGT-ATC-3′

Internal control PCR targeting phocid herpesvirus DNA			

Phocid herpes virus	5′-GGG-CGA-ATC-ACA-GAT-TGA-ATC-3′	5′-GCG-GTT-CCA-AAC-GTA-CCA-A-3′	5′-TTT-TTA-TGT-GTC-CGC-CAC-CAT-CTG-GATC-3′

**Table 2:** Median and mean cycle threshold (Ct) values of pathogens that were detected by PCR.

Detected pathogen	Median Ct-value	Mean Ct-value	Standard deviation
*Salmonella* spp.	27	27	—
*Shigella* spp./EIEC^1^	17.5	18	3.0
*Shigella* spp./EIEC^2^	13.5	14.7	2.7
*Shigella* spp./EIEC^3^	12.5	13.7	2.4
EPEC	22	21.4	4.0
STEC^4^	14	15.9	3.9
STEC^5^	19	18.2	4.3
EAEC	19	19.2	3.5
*Giardia duodenalis *	25.5	25.5	3.5
*Cryptosporidium parvum *	27	27.7	6.0
Norovirus G1	20	24.3	8.4
Norovirus G2	17	16.6	4.3
Astrovirus	30	30	—
Rotavirus	22	22	—
Sapovirus	32	32	—

^1^As detected by in-house PCR. ^2^As detected by RidaGene PCR targeting EHEC/EPEC/*Shigella* spp./EIEC. ^3^As detected by RidaGene PCR targeting ETEC and *Shigella* spp./EIEC. ^4^As detected by PCR targeting the stable toxin. ^5^As detected by PCR targeting the labile toxin.

**Table 3:** Ct-values in cases of multiple pathogen detections (in brackets). If both stable and labile toxin of STEC were detected, two Ct-values are given, otherwise only one. In case of *Shigella* spp./EIEC detections, three Ct-values are shown, reflecting the three applied PCR approaches. In several cases, low Ct-values are measured for more than one pathogen.

Case	Pathogen 1	Pathogen 2	Pathogen 3	Pathogen 4	Pathogen 5
1	*Shigella* spp./EIEC (Ct 13, 14, 16)	EPEC (Ct 18)	ETEC (Ct 13, 16)	EAEC (Ct 19)	Norovirus G1 (Ct 20)
2	*Salmonella* spp. (Ct 27)	EPEC (Ct 15)	Norovirus G2 (Ct 11)	Astrovirus (Ct 30)	—
3	EPEC (Ct 29)	EAEC (Ct 19)	Norovirus G2 (Ct 17)	—	—
4	ETEC (Ct 17, 20)	EAEC (Ct 19)	Norovirus G2 (Ct 14)	—	—
5	ETEC (Ct 13, 20)	EAEC (Ct 18)	Norovirus G2 (Ct 21)	—	—
6	*Shigella* spp./EIEC (Ct 12, 13, 14)	EPEC (Ct 21)	ETEC (Ct 21)	—	—
7	EPEC (Ct 17)	ETEC (Ct 20)	EAEC (Ct 19)	—	—
8	EPEC (Ct 20)	ETEC (Ct 13)	EAEC (Ct 21)	—	—
9	EPEC (Ct 16)	ETEC (Ct 13)	EAEC (Ct 22)	—	—
10	ETEC (Ct 15)	EAEC (Ct 15)	*Cryptosporidium parvum* (Ct 34)	—	—
11	EPEC (Ct 23)	ETEC (Ct 16, 21)	EAEC (Ct 22)	—	—
12	*Shigella* spp./EIEC (Ct 18, 20, 21)	EPEC (Ct 26)	—	—	—
13	EPEC (Ct 28)	*Giardia duodenalis* (Ct 28)	—	—	—
14	EPEC (Ct 21)	ETEC (Ct 17)	—	—	—
15	EPEC (Ct 25)	ETEC (Ct 12)	—	—	—
16	EPEC (Ct 22)	Norovirus G2 (Ct 17)	—	—	—
17	ETEC (Ct 11, 18)	EAEC (Ct 14)	—	—	—
18	ETEC (Ct 15)	EAEC (Ct 23)	—	—	—
19	EAEC (Ct 18)	*Cryptosporidium parvum* (Ct 22)	—	—	—
20	EPEC (Ct 22)	Sapovirus (Ct 32)	—	—	—
21	*Shigella* spp./EIEC (Ct 12, 23, 18)	EPEC (Ct 19)	—	—	—
22	EPEC (Ct 22)	ETEC (Ct 19, 19)	—	—	—
23	EPEC (Ct 21)	ETEC (Ct 24, 29)	—	—	—

**Table 4:** Age, gender, nationality, sites of deployment, and previous stays in high-endemicity regions for diarrhea-associated pathogens of diarrhea patients with bacterial, viral, and parasitic infections.

	Detected pathogen groups				
	Bacteria	Viruses	Parasites	No detected pathogen	Multiple infections
Age					
<30 years	10/15	6/15	0/15	2/15	5/15
30–50 years	10/15	3/15	1/15	3/15	8/15
>50 years	2/2	1/2	0/2	0/2	2/2
No data	12/17	2/17	2/17	4/17	7/17
Gender					
Male	31/44	11/44	3/44	7/44	20/44
Female	3/5	1/5	0/5	2/5	2/5
Nationality					
Austria	1/1	1/1	0/1	0/1	1/1
Belgium	7/9	2/9	1/9	1/9	3/9
Colombia	1/1	0/1	0/1	0/1	0/1
Germany	17/25	8/25	0/25	4/25	12/25
Greece	3/3	0/3	1/3	0/3	3/3
Ireland	0/1	0/1	0/1	1/1	0/1
Italy	0/1	0/1	0/1	1/1	0/1
Portugal	0/1	0/1	0/1	1/1	0/1
Romania	0/1	1/1	0/1	0/1	0/1
Spain	4/4	0/4	0/4	0/4	2/4
Spain/Bolivia	0/1	0/1	0/1	1/1	0/1
No data	1/1	0/1	1/1	0/1	1/1
Site of deployment					
Koulikoro	31/41	7/41	3/41	6/41	19/41
Koulikoro and Bamako	1/2	1/2	0/2	1/2	1/2
Bamako	0/1	0/1	0/1	1/1	0/1
Bapho	2/4	4/4	0/4	0/4	2/4
No data	0/1	0/1	0/1	1/1	0/1
Previous stays in high endemicity settings					
Yes	8/13	3/13	1/13	2/13	4/13
No	26/36	9/36	2/36	7/36	18/36

**Table 5:** Mode of food intake of diarrhea patients with bacterial, viral, and parasitic infections.

	Detected pathogen groups				
	Bacteria	Viruses	Parasites	No detected pathogen	Multiple infections
Field kitchen only	12/15	1/15	1/15	2/15	6/15
Field kitchen and field rations	1/1	0/1	0/1	0/1	1/1
Field kitchen and field rations and outdoor facility	2/4	0/4	1/4	1/4	2/4
Field kitchen and field rations and hotel and outdoor facility	2/2	0/2	0/2	0/2	0/2
Field kitchen and field rations and hotel and restaurant	1/1	1/1	0/1	0/1	1/1
Field kitchen and hotel	3/5	1/5	0/5	2/2	2/5
Field kitchen and hotel and outdoor facility	3/3	2/3	1/3	0/3	3/3
Field kitchen and outdoor facility	3/5	2/5	0/5	0/5	1/5
Field kitchen and restaurant	2/3	0/3	0/3	1/3	1/3
Hotel	4/7	5/7	0/7	1/7	4/7
Hotel and outdoor facility	0/1	0/1	0/1	1/1	0/1
Restaurant	1/1	0/1	0/1	0/1	1/1
No data	0/1	0/1	0/1	1/1	0/1

**Table 6:** Stool frequency and consistency in diarrhea patients with bacterial, viral, and parasitic infections.

	Detected pathogen groups				
	Bacteria	Viruses	Parasites	No detected pathogen	Multiple infections
Stool frequency					
1-2 stools per day	8/11	5/11	1/11	1/11	7/11
≥3 stools per day	23/34	6/34	2/34	7/34	14/34
No data	3/4	1/4	0/4	1/4	1/4
Stool consistency as described by the patient					
Watery	18/26	8/26	3/26	3/26	12/26
Mushy	4/5	0/5	0/5	1/5	2/5
Changing	2/3	0/3	0/3	1/3	2/3
Watery/mushy	4/5	1/5	0/5	1/5	2/5
Watery/changing	0/1	1/1	0/1	0/1	0/1
Slimy/changing	0/1	0/1	0/1	1/1	0/1
No data	6/8	2/8	0/8	2/8	4/8
Stool consistency as observed by the technical assistant					
Hard	10/13	1/13	1/13	3/13	6/13
Watery	11/14	2/14	1/14	1/14	7/14
Mushy	12/17	9/17	0/17	1/17	8/17
Slimy	1/2	0/2	1/2	1/2	1/2
No data	0/3	0/3	0/3	3/3	0/3

**Table 7:** Accompanying symptoms in diarrhea patients with bacterial, viral, and parasitic infections. n.o. = not observed.

	Detected pathogen groups				
	Bacteria	Viruses	Parasites	No detected pathogen	Multiple infections
Accompanying symptoms in all patients					
Nausea/vomiting	8/15	7/15	0/15	4/15	6/15
Cramps	15/20	6/20	2/20	2/20	10/20
Abdominal pain	19/29	8/29	2/29	5/29	10/29
Flatulence	9/13	4/13	1/13	2/13	6/13
Bloody diarrhea	1/1	0/1	0/1	0/1	0/1
No data	9/12	1/12	1/12	3/12	7/12
Fever					
Yes	2/3	1/3	0/3	1/3	2/3
No	32/46	11/46	3/46	8/46	20/46

Accompanying symptoms in patients with ≥ 3 stools per day (*n* = 34)					
Nausea/vomiting	4/9	2/9	0/9	4/9	2/9
Cramps	12/16	3/16	2/16	2/16	8/16
Abdominal pain	15/22	5/22	2/22	4/22	8/22
Flatulence	6/9	3/9	1/9	2/9	5/9
Bloody diarrhea	1/1	0/1	0/1	0/1	0/1
No data	5/7	0/7	0/7	2/7	3/7
Fever					
Yes	2/3	1/3	0/3	1/3	2/3
No	21/31	5/31	2/31	6/31	12/31

Accompanying symptoms in patients with only 1 detected pathogen and assessed symptom data (*n* = 16)					
Nausea/vomiting	EPEC (1/2), *Shigella* spp./EIEC (1/2)		Norovirus G2 (2/2), Norovirus G1 (1/2)	*Cryptosporidium parvum* (1/1)	
Cramps	*Shigella* spp./EIEC (2/2), STEC (3/4)		Norovirus G1 (2/2)	n.o.	
Abdominal pain	EAEC (2/2), EPEC (2/2), *Shigella* spp./EIEC (2/2), STEC (3/4)		Norovirus G1 (2/2), Norovirus G2 (2/2)	*Cryptosporidium parvum* (1/1)	
Flatulence	EAEC (1/2), *Shigella* spp./EIEC (1/2), STEC (1/4)		Rotavirus (1/1), Norovirus G1 (1/2)	n.o.	
Bloody diarrhea	*Shigella* spp./EIEC (1/2)		n.o.	n.o.	
Fever					
Fever was not observed.					

**Table 8:** Antimalarial prophylaxis, hygiene counseling, vaccination against cholera and typhoid fever, and medication of diarrhea patients with bacterial, viral, and parasitic infections.

	Detected pathogen groups				
	Bacteria	Viruses	Parasites	No detected pathogen	Multiple infections
Malaria prophylaxis					
Doxycycline	7/10	2/10	1/10	2/10	3/10
Atovaquone/proguanil	18/26	8/26	1/26	4/26	12/26
Mefloquine	8/9	2/9	1/9	0/9	6/9
Switch atovaquone/proguanil to doxycycline	1/1	0/1	0/1	0/1	1/1
None	0/1	0/1	0/1	1/1	0/1
No data	0/2	0/2	0/2	2/2	0/2
Vaccines					
Typhoid fever	23/32	7/32	3/32	5/32	15/32
Cholera and typhoid fever	7/10	1/10	0/10	3/10	4/10
No data	4/7	4/7	0/7	1/7	3/7
Medication					
Antibiotics	1/3	0/3	0/3	2/3	0/3
No antibiotics or uncertain	33/46	12/46	3/46	7/46	22/46
Hygiene counseling					
Yes	28/39	7/39	3/39	7/39	17/39
No	1/1	0/1	0/1	0/1	0/1
No data	5/9	5/9	0/9	2/9	5/9
